# Coexisting adenomyosis in endometriosis: Similar pain and quality of life, with longer bleeding and bowel symptoms—A comparative cross‐sectional study

**DOI:** 10.1002/ijgo.70988

**Published:** 2026-04-17

**Authors:** Mariana Matzenbacher Brunoro, Ana Clara Pimenta Servidoni, Sofia Di Nur Wakabayashi, Camila Rebeque Dagola, Marina Paula Andres, Mauricio Simoes Abrao

**Affiliations:** ^1^ Gynecologic Division, BP—A Beneficência Portuguesa de São Paulo São Paulo SP Brazil; ^2^ Faculdade Santa Marcelina São Paulo Brazil; ^3^ Disciplina de Ginecologia, Departamento de Obstetrícia e Ginecologia, Faculdade de Medicina FMUSP Universidade de São Paulo São Paulo Brazil

**Keywords:** abnormal uterine bleeding, adenomyosis, endometriosis, menstrual bleeding, quality of life, symptoms

## Abstract

**Objective:**

To compare pain, menstrual flow, and quality of life (QoL) between women undergoing surgery for endometriosis only compared with women with endometriosis and coexisting adenomyosis.

**Methods:**

A cross‐sectional study, set in two tertiary hospitals, of reproductive‐aged women (18–50 years) with a preoperative imaging diagnosis of endometriosis and/or adenomyosis and clinical indication for laparoscopy; all diagnoses were confirmed histologically. Groups were divided into pure endometriosis (*n* = 60) and endometriosis plus adenomyosis (*n* = 59) groups. Women responded to standardized questionnaires about demographics, gynecologic, history, and symptoms. Pain was graded by visual analog scale. Menstrual blood loss was assessed using the Pictorial Blood Loss Assessment Chart (PBAC). QoL was measured by the 36‐Item Short Form Survey (SF‐36) questionnaire. Surgical findings and the American Association of Gynecologic Laparoscopists classification were recorded.

**Main Results:**

We measured pain intensity, PBAC score and bleeding duration, and SF‐36 domains. The adenomyosis group was older (42.3 vs. 37.7 years, *P* = 0.001), reported longer menstrual bleeding duration (10.5 vs. 6.7 days), and had more cyclical bowel changes (55% vs. 28%). The groups did not differ in overall pain scores, infertility, PBAC categories, or SF‐36 domains. In multivariable analysis, older age at laparoscopy (odds ratio [OR], 1.15 [95% confidence interval (CI), 1.06–1.26]; *P* = 0.002) and longer menstrual bleeding duration (OR, 1.09 [95% CI, 1.02–1.17]; *P* = 0.010) independently increased the odds of adenomyosis; partial cul‐de‐sac obliteration was also associated with adenomyosis, whereas complete obliteration was more frequent in pure endometriosis.

**Conclusion:**

In patients with endometriosis, coexisting adenomyosis did not add to pain or worsen QoL but was linked to longer bleeding and increased bowel symptoms. Management should be individualized and guided primarily by symptom burden and life impact rather than imaging labels alone.

## 1 INTRODUCTION

Endometriosis and adenomyosis are prevalent, often coexisting disorders that contribute substantially to menstrual and pain‐related morbidity and impaired quality of life (QoL) in reproductive‐aged women.^1^, ^2^ Although adenomyosis is frequently presumed to exacerbate symptom burden when present with endometriosis, evidence remains inconsistent, and many studies lack histological confirmation or validated measures for bleeding and health‐related QOL (HRQoL). Both conditions remain challenging to diagnose because their detection relies on recognition of characteristic symptoms, while confirmation depends on high‐quality imaging, such as transvaginal ultrasound (TVUS) or magnetic resonance imaging (MRI).^3^, ^4^

Evidence shows a strong association between adenomyosis and endometriosis, with co‐occurrence reported in 13% to 89% of women with endometriosis.^5^ The coexistence of both disorders is associated with more severe clinical manifestations, including heavy menstrual bleeding, dysmenorrhea, and chronic pelvic pain, as well as dyspareunia, dyschezia, and, less frequently, infertility and urinary symptoms.^5^, ^6^ In addition to physical complaints, both disorders negatively affect sexual function and HRQoL.^7^

We aimed to compare pain, menstrual bleeding, and HRQoL between women with histologically confirmed endometriosis only versus women with endometriosis and concomitant adenomyosis. We also evaluated surgical findings and independent predictors of adenomyosis. We hypothesized that adenomyosis would not necessarily translate into greater pain or worse HRQoL among women already selected for surgery because of significant symptoms.

## 2 METHODS

### 2.1 Study design and setting

A cross‐sectional study was conducted at Hospital das Clínicas, University of São Paulo (HCFMUSP; a public hospital) and at A Beneficência Portuguesa de São Paulo Hospital (BP; a private hospital). Recruitment and data collection occurred from January 2017 to December 2023. The study was interrupted between 2020 and 2022, during the COVID‐19 pandemic.

### 2.2 Eligibility criteria

Women aged 18 to 50 years with preoperative imaging suggestive of endometriosis and/or adenomyosis by transvaginal ultrasound and/or pelvic MRI^8^, ^9^ were included. Clinical indications for laparoscopy comprised pelvic pain or abnormal uterine bleeding not improved after 6 months of hormonal treatment, infertility (inability to conceive after 12 months without contraception), histopathological confirmation of endometriosis in the removed lesions, and/or adenomyosis after hysterectomy following surgery.

All women in the adenomyosis plus endometriosis group underwent hysterectomy, while in the pure endometriosis group, the absence of adenomyosis was defined by imaging or hysterectomy. The histological analysis was defined as the gold standard. Adenomyosis was defined histologically as the presence of ectopic endometrial glands and stroma within the myometrium, typically located at least 2.5 mm from the endometrial–myometrial junction and associated with surrounding smooth muscle hyperplasia.^10^, ^11^

Exclusion included incomplete clinical data, uterine or endometrial malignancy, dominant fibroid ≥7 cm, and absence of histopathological confirmation of either condition.

### 2.3 Ethical approval and consent

All participants provided written informed consent. The study adhered to the Declaration of Helsinki and received approval from institutional ethics committees (HCFMUSP, No. 5.537.657/2022; BP, No. 5.652.700/2022).

### 2.4 Data collection and definitions

A standardized self‐administered questionnaire was applied during preoperative consultation, up to 3 months from the surgical procedure. The questionnaire captured demographics; obstetric‐gynecologic history; dysmenorrhea, deep dyspareunia, and noncyclical pelvic pain; cyclic dyschezia and dysuria; and infertility and prior infertility treatment. Pain intensity was recorded on a 0 to 10 numerical rating scale^12^ (0 = no pain; 10 = worst possible).^12^, ^13^ Menstrual blood loss was quantified using the Pictorial Blood Loss Assessment Chart (PBAC).^14^ The PBAC is a visual, self‐reported, and simple method to quantify menstrual blood loss, and it is validated in Portuguese. The PBAC should be filled out each day of the patient's menstrual bleeding, with the number of pads and/or tampons used, its saturation, and the number of blood cloths. After that, a standardizing punctuation is given, and the menstrual flow is categorized as normal (<100) or heavy (≥100). Participants also rated bleeding subjectively (mild/normal/high/absent). HRQoL was assessed by 36‐Item Short Form Survey (SF‐36)^15^, ^16^ among eight domains, validated to Portuguese. The menstrual flow was recorded during one typical menstrual cycle, regardless of the use of hormonal therapy.

### 2.5 Imaging and surgery

Preoperative diagnosis of adenomyosis was based on the Morphological Uterus Sonographic Assessment (MUSA) criteria.^8^, ^17^ Briefly, adenomyosis was characterized on TVUS or MRI by the presence of direct and indirect sonographic features, including myometrial cysts, hyperechoic islands, echogenic subendometrial lines and buds, and translesional vascularity on Doppler imaging, and globular uterus, asymmetrical myometrial thickening, fan‐shaped shadowing, heterogeneous myometrial echotexture, and abnormalities of the junctional zone. The diagnosis was established when one or more of these characteristic features were identified. All adenomyosis cases were confirmed histologically after hysterectomy.

For endometriosis, we followed the International Deep Endometriosis Analysis (IDEA) group consensus.^18^ Endometriosis was identified by the presence of specific sonographic features depending on lesion location. A systematic evaluation of the uterus and adnexa, assessment of soft markers (e.g. site‐specific tenderness, ovarian mobility), and dynamic assessment of the sliding sign were performed in accordance with IDEA recommendations. All patients who underwent TVUS had an oral laxative intake (sodium picosulfate) a day before (at 10 am and 4 pm) and a rectal enema (120 mL of sodium diphosphate) 1 h before the examination. Bowel preparation was routinely used for better visualization of rectosigmoid lesions. Ultrasounds were performed by experienced radiologists with >10 years of experience with this method using a Voluson E10 (GE Healthcare) equipped with a high‐frequency endovaginal transducer. Mapping and staging of endometriosis were performed according to established protocols.^19^

MRI was performed with 1.5 T scanners (Aerea, Siemens Healthcare, GmbH) using a body‐array coil. The protocol included single‐shot fast spin echo (SSFSE) two‐dimensional T2‐weighted sagittal (TE = 75 ms, TR = 3200 ms, thickness = 4.0 mm, pixel spacing = 44.8 × 28.5 cm); SSFSE two‐dimensional T2‐weighted axial (TE = 119 ms, TR = 4229 ms, thickness = 4.0 mm, pixel spacing = 34.6 × 22.0 cm); SSFSE three‐dimensional T2‐weighted (TE = 130 ms, TR = 1500 ms, slice thickness = 1.2 mm, pixel spacing = 44.8 × 28.5 cm), and axial two‐dimensional T1 gradient echo, with fat saturation, VIBE / LAVA sequence (TE = 6.8 ms, TR = 2.4 ms, thickness = 2 mm, pixel spacing = 44.0 × 28.0 cm) acquired before and after the administration of the paramagnetic agent (Dotaren) (0.1 mL/kg body weight). Immediately before the beginning of the examination, an ampoule of n‐butylscopolamine was administered intravenously to decrease the peristaltic movement artifacts. Routinely, a vaginal gel (50 mL) was also introduced to distend the vaginal fornix. No rectal gel was used. Experienced (10 years) radiologists evaluated MRI findings.

During laparoscopy, the pelvis was inspected and suspected endometriosis lesions were excised, following current guidelines.^20^ Procedures were performed by the same surgical team, highly experienced in endometriosis treatment. The posterior cul‐de‐sac was inspected and classified as no obliteration, partial obliteration, or complete obliteration based on the degree of adhesions and distortion of the rectouterine pouch, as previously described in surgical studies of deep endometriosis.^21^

Radiologists and surgeons were not blinded to suspected diagnoses, reflecting routine practice. At laparoscopy, endometriosis location, severity, and American Association of Gynecologic Laparoscopists (AAGL)^22^ surgical score/stage were recorded.

### 2.6 Outcomes

Primary outcomes were pain intensity, measured using the visual analog scale (VAS) from 0 to 10, PBAC^14^ score, bleeding duration, and SF‐36^15^ domain scores. Secondary outcomes included pain symptoms (dysmenorrhea, dyspareunia, noncyclical pelvic pain, cyclic dyschezia or bowel changes, cyclic dysuria, or urinary changes), other cyclic bowel symptoms (such as rectal bleeding, constipation, or diarrhea), cyclic urinary symptoms (pollakiuria, urgency, or urinary bleeding), infertility, distribution of disease (including cul‐de‐sac obliteration), and factors associated with adenomyosis.

### 2.7 Sample size and power

Based on prior data comparing dysmenorrhea between endometriosis alone versus coexisting adenomyosis,^23^ we estimated 58 participants per group to detect a clinically relevant difference with *α* = 0.05 and 80% power. Our final sample met this target for primary between‐group comparisons but remained underpowered for small effects and subgroup analyses.

### 2.8 Statistical analysis

The distribution of continuous variables was assessed using the Shapiro–Wilk test and visual inspection of histograms and Q–Q plots. Variables with normal distribution were expressed as mean ± standard deviation, whereas nonnormally distributed variables were reported as median and interquartile range. When multiple comparisons were performed, appropriate adjustments were considered to control for type I error. Categorical variables were compared using *χ* ^2^ or Fisher exact tests. To evaluate changes in outcomes over time, including pain assessed by the VAS and the domains of the SF‐36 questionnaire, linear regression models were performed comparing the pure endometriosis group and the endometriosis plus adenomyosis group. In these models, study group was included as a fixed effect. Variables with a *P* value ≤0.20 were considered potential confounders and selected for inclusion in the initial adjusted model. Subsequently, only variables that were significant in the univariate analysis were retained for the multivariable model. A backward elimination procedure was then applied, whereby variables with the highest *P* values were sequentially removed until only statistically significant variables remained in the final model. Results are presented as β coefficients with their corresponding 95% confidence intervals (CIs).

The use of hormonal treatment and presence of myomas were included as covariate in the analysis. Odds ratios (ORs) with 95% CIs were reported. Analysis used SPSS version 25 (IBM). Missing data were assessed for all variables included in the analysis. The proportion of missing values for each variable was quantified and found to be low. Therefore, analyses were conducted using a complete‐case approach, including only observations with available data for the variables of interest. Given the limited proportion of missing data, this approach was considered unlikely to introduce significant bias.

## 3 RESULTS

### 3.1 Cohort and baseline characteristics

A total of 206 consecutive patients were initially screened for eligibility according to the study protocol. Of these, 170 patients met the inclusion criteria. After surgical and histological confirmation, 119 patients were included in the final analysis (59 to the adenomyosis plus endometriosis group and 60 to the pure endometriosis group) (Figure 1). Patients in the adenomyosis plus endometriosis group were older (42.3 ± 5.2 years vs. 37.7 ± 6.3 years; *P* < 0.001; Table 1) and were more likely to come from the private health care sector than the pure endometriosis group (49.2% vs. 25.0%; *P* = 0.006). Pain scores (dysmenorrhea, dyspareunia, noncyclical pelvic pain) did not differ between groups. Other intestinal symptoms were more prevalent in the adenomyosis plus endometriosis compared with the pure endometriosis group (55.0% vs. 28.0%; *P* < 0.001), mainly represented by cyclic diarrhea (64.0% vs. 53.0%, respectively).

**FIGURE 1:**
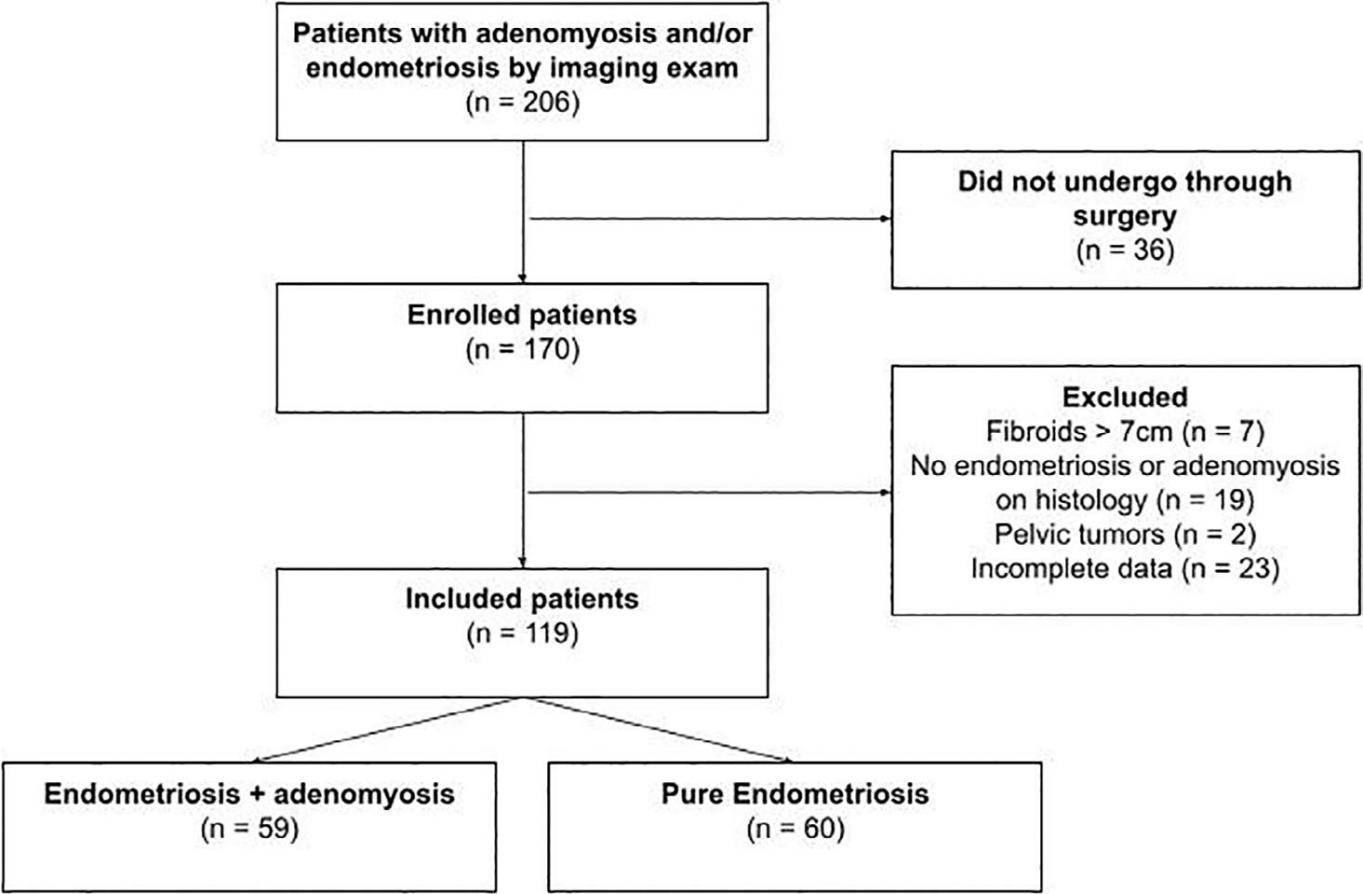
Flow chart of included patients.

**TABLE 1:** Characteristics of included patients.

Characteristics	Adenomyosis plus endometriosis (*n* = 59)	Pure endometriosis (*n* = 60)	*P* value
Age, year	42.3 ± 5.2	37.7 ± 6.3	**<0.001** ^a^
BMI, kg/m^2^	27.6 ± 4.3	27.1 ± 5.0	0.4^a^
Sector			**0.006** ^b^
Public	30 (50.8)	45 (75.0)	
Private	29 (49.2)	15 (25.0)	
Dysmenorrhea (VAS)	7.9 ± 2.9	7.9 ± 2.9	>0.98^a^
Dyspareunia (VAS)	6.2 ± 3.8	6.3 ± 3.6	0.91^a^
Acyclic pelvic pain (VAS)	5.7 ± 3.9	5.8 ± 3.5	0.93^a^
Cyclic dyschezia (VAS)	5.3 ± 4.3	5.1 ± 4.1	0.79^a^
Other cyclical intestinal changes	31 (55.0)	17 (28.0)	**0.006** ^b^
Diarrhea	16 (64.0)	23 (53.0)	0.192^b^
Obstipation	5 (20.0)	13 (30.0)	0.07^c^
Rectal bleeding	4 (16.0)	5 (12.0)	>0.001^c^
Cyclic dysuria (VAS)	1.9 ± 3.2	2.8 ± 3.2	0.11^a^
Other cyclic urinary alteration	19 (33.0)	14 (24.0)	0.3^b^
Infertility			0.4^b^
No	27 (46.0)	22 (37.0)	
Yes	15 (25.0)	20 (33.0)	
Not trying to conceive	17 (29.0)	16 (27.0)	
Not reported	0 (0.0)	2 (3.3)	
Prior treatment for infertility	44 (75.0)	51 (85.0)	0.3^b^

*Note*: Numerical data are expressed as mean ± standard deviation and categorical data as number (percentage). Significant comparisons are highlighted in bold.

Abbreviations: BMI, body mass index; VAS, visual analog scale.

^a^ Student *t*‐test.

^b^ *χ* ^2^ test.

^c^ Fisher exact test.

### 3.2 Menstrual bleeding and fibroids

Women with adenomyosis plus endometriosis reported longer bleeding duration (11.1 ± 10.0 vs. 6.7 ± 5.6 days; *P* = 0.005). Subjective flow was evaluated as high in a large proportion of patients in both groups (65.0% in the adenomyosis plus endometriosis group vs. 47.0% in the pure endometriosis group). Menstrual flow evaluated as high by PABC score was prevalent in both groups with no significant difference between them (75.0% vs. 61.0%, respectively).

The use of hormonal treatment was reported by 53.3% of patients in the pure endometriosis group and 61.6% in the adenomyosis plus endometriosis group (*P* = 0.31, Table 2), with dienogest being the most frequently reported (41.0% vs. 41.0%), followed by progestogens (25.4% vs. 16.7%) and combined contraceptives (16.0% vs. 14.0%).

**TABLE 2:** PABC score, menstrual flow characteristics, and fibroids evaluation on imaging examinations.

Characteristic	Adenomyosis plus endometriosis (*n* = 59)	Pure endometriosis (*n* = 60)	*P* value
PBAC score*	182.5 (5.00–476.0)	261.0 (76.0–660.0)	0.4^a^
PBAC volume*			0.3^b^
Normal (<100)	11 (22.0)	18 (33.0)	
High (≥100)	38 (75.0)	33 (61.0)	
Absent	2 (3.9)	3 (5.6)	
Subjective intensity of menstrual* flow			0.2^b^
Mild	2 (3.7)	7 (12.0)	
Normal	10 (19.0)	16 (28.0)	
High	35 (65.0)	27 (47.0)	
Absent	7 (13.0)	7 (12.0)	
Length of menstrual flow, days*	11.1 ± 10.0	6.7 ± 5.6	**0.005** ^a^
Current hormonal treatment	32 (53.3)	37 (61.6)	0.31^b^
Dienogeste	13 (41.0)	15 (41.0)	
Gestrinone	1 (3.1)	0 (0.0)	
Combined	5 (16.0)	5 (14.0)	
Progestogen	8 (25.4)	6 (16.7)	
IUD‐levonorgestrel	2 (6.3)	4 (11.0)	
GnRH agonist	2 (6.3)	2 (5.4)	
Other	1 (3.1)	5 (14.0)	
Myomas	37 (63.0)	24 (40.0)	0.022^b^
No.	2.5 ± 2.0	2.2 ± 1.7	0.2^a^
Size of the greatest, cm	2.3 ± 1.9	3.1 ± 2.3	0.2^a^
Mean size, cm	2.1 ± 1.4	2.3 ± 1.0	0.2^a^
Total number of myomas, *n*	69	48	
Location			0.11^b^
Subserosal	19 (27.5)	9 (18.8)	
Intramural	43 (62.6)	26 (54.2)	
Submucosal	3 (4.3)	5 (10.4)	
Ignored	4 (5.8)	8 (16.7)	

*Note*: Numerical data is expressed as median (interquartile interval) or mean ± standard deviation and categorical data as number (percentage). Significant comparisons are highlighted in bold.

Abbreviations: IUD, intrauterine device; PBAC, Pictorial Blood Loss Assessment Chart.

^a^ Student *t*‐test.

^b^ *χ* ^2^ test.

* Hormonal treatment was included as covariate in the analysis.

Fibroids were observed in 63% of patients in the adenomyosis plus endometriosis group and 40% of patients in the pure endometriosis group (*P* = 0.022; Table 2). However, the number, size of the greatest myoma, and location were similar between both groups.

### 3.3 Surgical findings

In subgroup analysis of women with confirmed endometriosis at surgery (excluding 18 patients with pure adenomyosis), anatomic distribution and AAGL score/stage were comparable between groups. Cul‐de‐sac obliteration differed, with the pure endometriosis group having more complete obliteration (23.0%) and the adenomyosis plus endometriosis group having more partial obliteration (17.0%, *P* = 0.047; Table 3).

**TABLE 3:** Endometriosis location according to AAGL classification during surgery (excluding patients without endometriosis).

Surgical findings	Adenomyosis plus endometriosis* (*n* = 41)	Pure endometriosis (*n* = 60)	*P* value
Endometriosis			
Superficial	15 (38.0)	21 (35.0)	0.4^b^
Deep			
Retrocervical	24 (58.0)	36 (59.7)	0.2^b^
Vagina	4 (9.8)	7 (11.7)	0.7^b^
Bladder	0 (0.0)	4 (6.7)	0.2^b^
Rectovaignal septum	0 (0.0)	5 (8.3)	0.2^b^
Rectosigmoid	19 (46.0)	33 (55.0)	0.3^b^
Small Bowel	4 (9.7)	3 (5.0)	0.4^b^
Appendix	2 (4.9)	8 (13.0)	0.3^b^
Right ovary	12 (29.7)	17 (28.4)	>0.9^b^
Left ovary	6 (14.4)	18 (30.0)	0.073^b^
Right ureter	1 (2.4)	3 (5.0)	0.9^b^
Left ureter	5 (12.0)	6 (10.0)	>0.9^b^
Left uterine tube	4 (9.8)	7 (11.7)	0.4^b^
Right uterine tube	5 (12.1)	5 (8.4)	0.6^b^
Cul‐de‐sac obliteration			**0.047** ^b^
Absent	30 (73.0)	43 (72.0)	
Partial	7 (17.0)	3 (5.0)	
Complete	4 (9.8)	14 (23.0)	
AAGL score	15.0 ± 9.1	17.9 ± 13.2	0.5^a^
AAGL stage			0.8^b^
I	12 (29.0)	20 (33.0)	
II	7 (17.0)	9 (15.0)	
III	8 (20.0)	11 (18.0)	
IV	13 (22.0)	20 (33.0)	

*Note*: Numerical data are expressed as mean ± standard deviation and categorical data as number (percentage). Significant comparisons are highlighted in bold.

Abbreviation: AAGL, American Association of Gynecologic Laparoscopists.

^a^ Student *t*‐test.

^b^ *χ* ^2^ test.

* Excluding 18 patients without endometriosis.

### 3.4 Quality of life

SF‐36^15^ domains were similarly impaired in both groups, with the lowest medians in physical and emotional role limitation domains and bodily pain, and the highest in social functioning. No domain showed a difference between groups (Table 4; Figure 2).

**TABLE 4:** QoL evaluated using SF‐36 questionnaire.

SF‐36 domains	Adenomyosis plus endometriosis (*n* = 59)	Pure endometriosis (*n* = 60)	*P* value
Physical functioning	54.0 ± 33.0	54.9 ± 30.9	<0.9^a^
Role physical	36.9 ± 44.1	27.0 ± 38.7	0.3^a^
Bodily pain	41.0 ± 29.1	40.8 ± 26.5	>0.9^a^
General health	46.2 ± 19.2	45.2 ± 17.5	0.9^a^
Vitality	41.7 ± 24.3	39.1 ± 22.2	0.7^a^
Social functioning	55.8 ± 14.5	59.8 ± 15.4	0.2^a^
Role emotional	41.7 ± 45.9	34.0 ± 43.0	0.5^a^
Mental health	44.2 ± 26.1	45.9 ± 19.2	0.6^a^

*Note*: Data are expressed as mean ± standard deviation.

^a^ Student *t*‐test.

**FIGURE 2:**
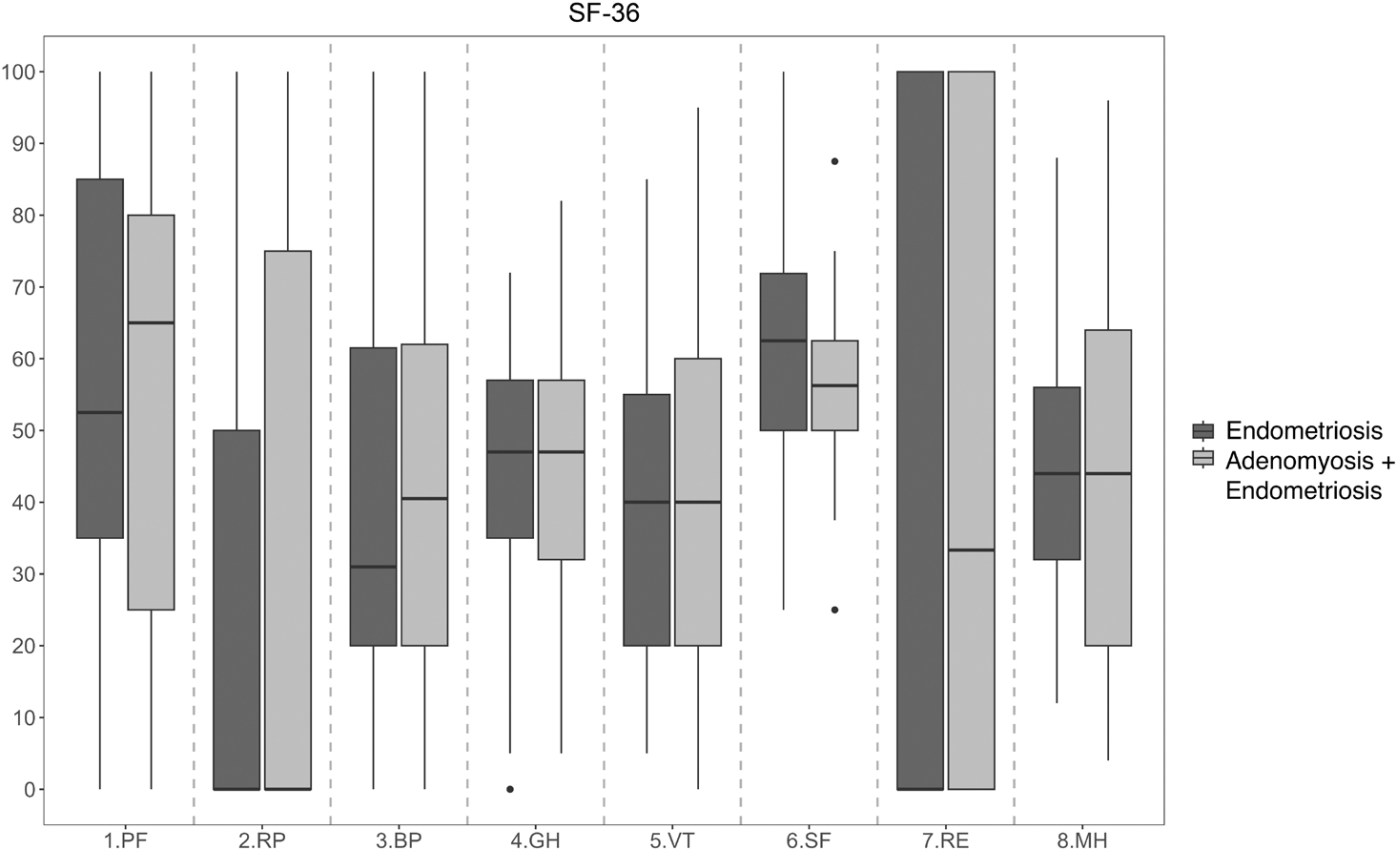
QoL in patients with pure endometriosis (*n* = 60) and endometriosis plus adenomyosis (*n* = 59). Data are expressed as means SF‐36 score for each domain. BP, bodily pain; GH, general health; MH, mental health; PF, physical functioning; QoL, quality of life; RE, role emotional; RP, role physical; SF, social functioning; SF‐36, 36‐Item Short Form Survey; VT, vitality.

### 3.5 Multivariable analysis

Independent associations with adenomyosis included older age (OR, 1.2 per year) and longer bleeding duration (OR, 1.1 per day). Partial cul‐de‐sac obliteration was associated with adenomyosis (OR, 7.4), while complete obliteration trended towards pure endometriosis (Table 5).

**TABLE 5:** Multiple logistic regression evaluating the risk of adenomyosis.

Characteristic	OR	95% CI	*P* value
Age	1.2	1.1–1.3	0.002
Length of menstrual bleeding	1.1	1.0–1.2	0.010
Cul‐de‐sac obliteration			
Absent	—		
Partial	7.4	1.5–46.8	0.020
Complete	0.5	0.1–1.9	0.300

*Note*: To evaluate changes in outcomes over time, including pain assessed by the visual analog scale and the domains of the SF‐36 questionnaire, linear regression models were performed comparing the pure endometriosis group and the endometriosis plus adenomyosis group. In these models, study group was included as a fixed effect. Variables with a *P* value ≤0.20 were considered potential confounders and selected for inclusion in the initial adjusted model. Subsequently, only variables that were significant in the univariate analysis were retained for the multivariable model. A backward elimination procedure was then applied, whereby variables with the highest *P* values were sequentially removed until only statistically significant variables remained in the final model. Results are presented as β coefficients with their corresponding 95% CIs.

Abbreviations: CI, confidence interval; OR, odds ratio; SF‐36, 36‐Item Short Form Survey.

## 4 DISCUSSION

In this surgical cohort, adenomyosis coexisting with endometriosis did not translate into higher pain intensity or worse HRQoL compared with endometriosis alone. However, adenomyosis was linked to longer menstrual bleeding and increased cyclic bowel changes. Although adenomyosis is frequently linked to dysmenorrhea and abnormal uterine bleeding, the incremental symptomatic burden may be less apparent in populations already selected for surgery because of clinically significant endometriosis‐related symptoms, as we observed in our population.^24^

The high prevalence of advanced endometriosis in this cohort likely reflects the characteristics of patients referred to tertiary referral centers for surgical treatment. In specialized endometriosis units, women undergoing surgery often present with stage III or IV disease American Society for Reproductive Medicine classification, frequently represented by deep infiltrating lesions and severe adhesions. Advanced disease is particularly associated with involvement of the posterior pelvic compartment, including the uterosacral ligaments, rectovaginal septum, and rectosigmoid colon. These lesions can lead to progressive fibrosis, adhesions, and distortion of pelvic anatomy, ultimately resulting in partial or complete obliteration of the pouch of Douglas. Surgical series have consistently reported cul‐de‐sac obliteration as a common finding in patients with deep infiltrating endometriosis and posterior compartment disease.

In the present study, although overall anatomical distribution and AAGL stage were similar between groups, the pattern of cul‐de‐sac obliteration differed. Complete obliteration was more frequent in patients with pure endometriosis, whereas partial obliteration was more commonly observed among patients with concomitant adenomyosis. Obliteration of the pouch of Douglas is considered an important marker of severe posterior compartment disease and is typically associated with dense adhesions between the posterior uterus, cervix, and anterior rectal wall. The finding that complete obliteration tended to occur more frequently in the pure endometriosis group may suggest that the severity of posterior compartment involvement is driven primarily by the endometriotic disease process itself rather than by the coexistence of adenomyosis.

Despite differences in bleeding patterns and bowel symptoms, both groups showed similarly impaired HRQoL among SF‐36 domains. Overall scores were low, particularly in domains related to role limitation due to physical and emotional problems and bodily pain. These findings are consistent with previous studies demonstrating that endometriosis substantially affects multiple aspects of patients' daily functioning and well‐being.^25^ Chronic pelvic pain, dysmenorrhea, dyspareunia, and gastrointestinal symptoms contribute to limitations in physical activities, reduced productivity, and psychological distress. Importantly, QoL impairment in endometriosis often persists even when differences in disease phenotype or anatomical extent are observed between patient subgroups. The absence of significant differences in HRQoL between groups may therefore reflect the already substantial symptom burden experienced by women referred for surgical evaluation.^26^ Previous research has shown that correlations between anatomical disease severity and symptom intensity are often weak, highlighting the multifactorial nature of symptom generation in endometriosis.^26^ Pain perception, central sensitization, psychological factors, and comorbid conditions may all influence the patient experience beyond the anatomical distribution of lesions.^27^ Consequently, the coexistence of adenomyosis may not necessarily lead to further measurable deterioration in QoL scores within a population already experiencing severe symptoms.

Fibroids were more prevalent in the adenomyosis plus endometriosis group, although their size, number, and location were similar between groups. This observation is consistent with the known overlap between adenomyosis and leiomyomas, both of which are estrogen‐dependent uterine disorders.^24^ The coexistence of fibroids represents a potential confounder when evaluating abnormal uterine bleeding and should be considered during clinical assessment and counseling. In patients presenting with heavy menstrual bleeding, clinicians should carefully evaluate the relative contributions of adenomyosis, fibroids, and other uterine pathologies when planning treatment strategies.

The finding that patients in the adenomyosis plus endometriosis group were older than those with isolated endometriosis aligns with known epidemiological patterns. Adenomyosis is typically diagnosed in women in their late reproductive years, with prevalence increasing after the age of 40, whereas endometriosis is more commonly diagnosed earlier, often in the third decade of life.^6^, ^24^, ^28^ Age may also influence menstrual characteristics, including changes in cycle regularity and bleeding volume, which could partially explain the longer bleeding duration observed in the adenomyosis group.^29^

This study has several strengths, including prospective data collection using validated symptom and QoL instruments, histological confirmation of adenomyosis, and detailed intraoperative characterization of endometriosis distribution. Nonetheless, several limitations should be acknowledged. The surgical nature of the cohort introduces inherent selection bias, as patients referred for laparoscopy often represent those with more severe or refractory symptoms. Additionally, the cross‐sectional design limits causal inference regarding symptom trajectories over time. Residual confounding factors, including variations in hormonal treatment exposure and the presence of fibroids, may also have influenced the observed associations. Finally, the sample size may have limited statistical power to detect subtle differences between groups.

From a clinical perspective, these findings reinforce the importance of individualized, symptom‐driven management strategies in patients with endometriosis. The presence of adenomyosis should be considered in the overall clinical context, particularly when abnormal uterine bleeding is a prominent complaint. However, treatment decisions should primarily focus on the patient's predominant symptoms, reproductive goals, and QoL impact rather than on the mere coexistence of adenomyosis.

## 5 CONCLUSION

In this surgical cohort of women with endometriosis, the coexistence of adenomyosis was not associated with higher pain scores or worse HRQoL. However, adenomyosis was associated with longer menstrual bleeding and more frequent cyclic bowel symptoms. The anatomical distribution and stage of endometriosis were similar between groups, although complete cul‐de‐sac obliteration was more frequent in patients with pure endometriosis. These findings suggest that, in patients already experiencing substantial disease burden, adenomyosis may contribute mainly to bleeding patterns rather than to additional pain or QoL impairment. Management should remain symptom‐driven and patient‐centered.

## AUTHOR CONTRIBUTIONS

Mariana Matzenbacher Brunoro: Study design; data collection; data analysis and interpretation; manuscript preparation and revision; patient recruitment. Ana Clara Pimenta Servidoni: Data collection; data analysis and interpretation; manuscript preparation. Sofia Di Nur Wakabayashi: Data collection; data analysis and interpretation; manuscript preparation. Camila Rebeque Dágola: Data collection; data analysis and interpretation; manuscript preparation. Marina Paula Andres: Study design; data analysis and interpretation; manuscript preparation; responsible surgeon. Mauricio Simoes Abrao: Study design; manuscript revision; responsible surgeon.

## FUNDING INFORMATION

No specific funding was received for this work.

## CONFLICT OF INTEREST STATEMENT

The authors declare no conflicts of interest.

## Data Availability

Deidentified data and analytic code are available from the corresponding author on reasonable request, subject to institutional policies.

## References

[1] Zondervan KT , Becker CM , Missmer SA . Endometriosis. N Engl J Med. 2020;382(13):1244‐1256. 10.1056/NEJMra1810764 32212520

[2] Bulun SE . Endometriosis. N Engl J Med. 2009;360(3):268‐279. 10.1056/NEJMra0804690 19144942

[3] Chapron C , Vannuccini S , Santulli P , et al. Diagnosing adenomyosis: an integrated clinical and imaging approach. Hum Reprod Update. 2020;26(3):392‐411. 10.1093/humupd/dmz049 32097456

[4] Van den Bosch T , Dueholm M , Leone FP , et al. Terms, definitions and measurements to describe sonographic features of myometrium and uterine masses: a consensus opinion from the Morphological Uterus Sonographic Assessment (MUSA) group. Ultrasound Obstet Gynecol. 2015;46(3):284‐298. 10.1002/uog.14806 25652685

[5] Pinzauti S , Lazzeri L , Tosti C , et al. Transvaginal sonographic features of diffuse adenomyosis in 18–30‐year‐old nulligravid women without endometriosis: association with symptoms. Ultrasound Obstet Gynecol. 2015;46(6):730‐736. 10.1002/uog.14834 25728241

[6] Vannuccini S , Petraglia F . Recent advances in understanding and managing adenomyosis. F1000Res. 2019;8:8. 10.12688/f1000research.17242.1 PMC6419978 30918629

[7] Moradi M , Parker M , Sneddon A , Lopez V , Ellwood D . Impact of endometriosis on women's lives: a qualitative study. BMC Womens Health. 2014;14:123. 10.1186/1472-6874-14-123 PMC4287196 25280500

[8] Harmsen MJ , Van den Bosch T , de Leeuw RA , et al. Consensus on revised definitions of Morphological Uterus Sonographic Assessment (MUSA) features of adenomyosis: results of modified Delphi procedure. Ultrasound Obstet Gynecol. 2022;60(1):118‐131. 10.1002/uog.24786 PMC9328356 34587658

[9] Bielen D , Tomassetti C , Van Schoubroeck D , et al. IDEAL study: magnetic resonance imaging for suspected deep endometriosis assessment prior to laparoscopy is as reliable as radiological imaging as a complement to transvaginal ultrasonography. Ultrasound Obstet Gynecol. 2020;56(2):255‐266. 10.1002/uog.21868 31503381

[10] Benagiano G , Brosens I , Habiba M . Structural and molecular features of the endomyometrium in endometriosis and adenomyosis. Hum Reprod Update. 2014;20(3):386‐402. 10.1093/humupd/dmt052 24140719

[11] Bird CC , McElin TW , Manalo‐Estrella P . The elusive adenomyosis of the uterus‐‐revisited. Am J Obstet Gynecol. 1972;112(5):583‐593. 10.1016/0002-9378(72)90781-8 5059589

[12] Andres MP , Riccio LGC , Abrao HM , Manzini MS , Braga L , Abrao MS . Visual analogue scale cut‐off point of seven represents poor quality of life in patients with endometriosis. Reprod Sci. 2024;31(4):1146‐1150. 10.1007/s43032-023-01406-6 38057531

[13] Bourdel N , Alves J , Pickering G , Ramilo I , Roman H , Canis M . Systematic review of endometriosis pain assessment: how to choose a scale? Hum Reprod Update. 2015;21(1):136‐152. 10.1093/humupd/dmu046 25180023

[14] Hald K , Lieng M . Assessment of periodic blood loss: interindividual and intraindividual variations of pictorial blood loss assessment chart registrations. J Minim Invasive Gynecol. 2014;21(4):662‐668. 10.1016/j.jmig.2014.01.015 24469275

[15] Campolina AG , Ciconelli RM . SF‐36 and the development of new assessment tools for quality of life. Acta Reumatol Port. 2008;33(2):127‐133. 18604180

[16] Ferreira PL . Development of the Portuguese version of MOS SF‐36. Part II—validation tests. Acta Medica Port. 2000;13(3):119‐127. 11026151

[17] Chapron C , Tosti C , Marcellin L , et al. Relationship between the magnetic resonance imaging appearance of adenomyosis and endometriosis phenotypes. Hum Reprod. 2017;32(7):1393‐1401. 10.1093/humrep/dex088 28510724

[18] Condous G , Gerges B , Thomassin‐Naggara I , et al. Non‐invasive imaging techniques for diagnosis of pelvic deep endometriosis and endometriosis classification systems: an international consensus statement. J Minim Invasive Gynecol. 2024;31(7):557‐573. 10.1016/j.jmig.2024.04.006 38819341

[19] Goncalves MO , Podgaec S , Dias JA , Gonzalez M , Abrao MS . Transvaginal ultrasonography with bowel preparation is able to predict the number of lesions and rectosigmoid layers affected in cases of deep endometriosis, defining surgical strategy. Hum Reprod. 2010;25(3):665‐671. 10.1093/humrep/dep433 20023291

[20] Becker CM , Bokor A , Heikinheimo O , et al. ESHRE guideline: endometriosis. Hum Reprod Open. 2022;2022(2):hoac009. 10.1093/hropen/hoac009 PMC8951218 35350465

[21] Revised American Society for Reproductive Medicine classification of endometriosis: 1996. Fertil Steril. 1997;67(5):817‐821. 10.1016/s0015-0282(97)81391-x 9130884

[22] Abrao MS , Andres MP , Miller CE , et al. AAGL 2021 endometriosis classification: an anatomy‐based surgical complexity score. J Minim Invasive Gynecol. 2021;28(11):1941‐1950.e1. 10.1016/j.jmig.2021.09.709 34583009

[23] Di Donato N , Seracchioli R . How to evaluate adenomyosis in patients affected by endometriosis? Minim Invasive Surg. 2014;2014:507230. 10.1155/2014/507230 PMC4146361 25197569

[24] Naftalin J , Hoo W , Pateman K , Mavrelos D , Foo X , Jurkovic D . Is adenomyosis associated with menorrhagia? Hum Reprod. 2014;29(3):473‐479. 10.1093/humrep/det451 24408315

[25] Garry R , Clayton R , Hawe J . The effect of endometriosis and its radical laparoscopic excision on quality of life indicators. BJOG. 2000;107(1):44‐54. 10.1111/j.1471-0528.2000.tb11578.x 10645861

[26] Dunselman GA , Vermeulen N , Becker C , et al. ESHRE guideline: management of women with endometriosis. Hum Reprod. 2014;29(3):400‐412. 10.1093/humrep/det457 24435778

[27] Christ JP , Yu O , Schulze‐Rath R , Grafton J , Hansen K , Reed SD . Incidence, prevalence, and trends in endometriosis diagnosis: a United States population‐based study from 2006 to 2015. Am J Obstet Gynecol. 2021;225(5):500.e1‐500.e9. 10.1016/j.ajog.2021.06.067 34147493

[28] Bordonné C , Puntonet J , Maitrot‐Mantelet L , et al. Imaging for evaluation of endometriosis and adenomyosis. Minerva Obstet Gynecol. 2021;73(3):290‐303. 10.23736/S2724-606X.21.04710-9 34008384

[29] Horne M , Martin KC . Abnormal uterine bleeding in women of reproductive age: getting to the diagnosis. Nurse Pract. 2025;50(9):17‐28. 10.1097/01.NPR.0000000000000351 40855589

